# Supplementary material for: Mentha pulegium L. (Pennyroyal, Lamiaceae) Extracts Impose Abortion or Fetal-Mediated Toxicity in Pregnant Rats; Evidenced by the Modulation of Pregnancy Hormones, MiR-520, MiR-146a, TIMP-1 and MMP-9 Protein Expressions, Inflammatory State, Certain Related Signaling Pathways, and Metabolite Profiling via UPLC-ESI-TOF-MS
Source: Toxins (Basel). 2022 May 16;14(5):347. doi: 10.3390/toxins14050347 (PMC9147109; doi:10.3390/toxins14050347)
Supplement: Supplementary file 1 [file toxins-14-00347-s001.zip › toxins-1713335-supplementary.pdf]

Supplementary Materials: *Mentha pulegium* L. (pennyroyal, Lamiaceae) extracts impose abortion or fetal-mediated toxicity in pregnant rats; evidenced by the modulation of pregnancy hormones, MiR-520, MiR-146a, TIMP-1 and MMP-9 protein expressions, inflammatory state, certain related signaling pathways, and metabolite profiling *via* UPLC-ESI-TOF-MS.

Amira A. El-Gazar, Ayat M. Emad, Ghada M. Ragab and **Dalia M. Rasheed**

Identification of caffeic acid derivatives

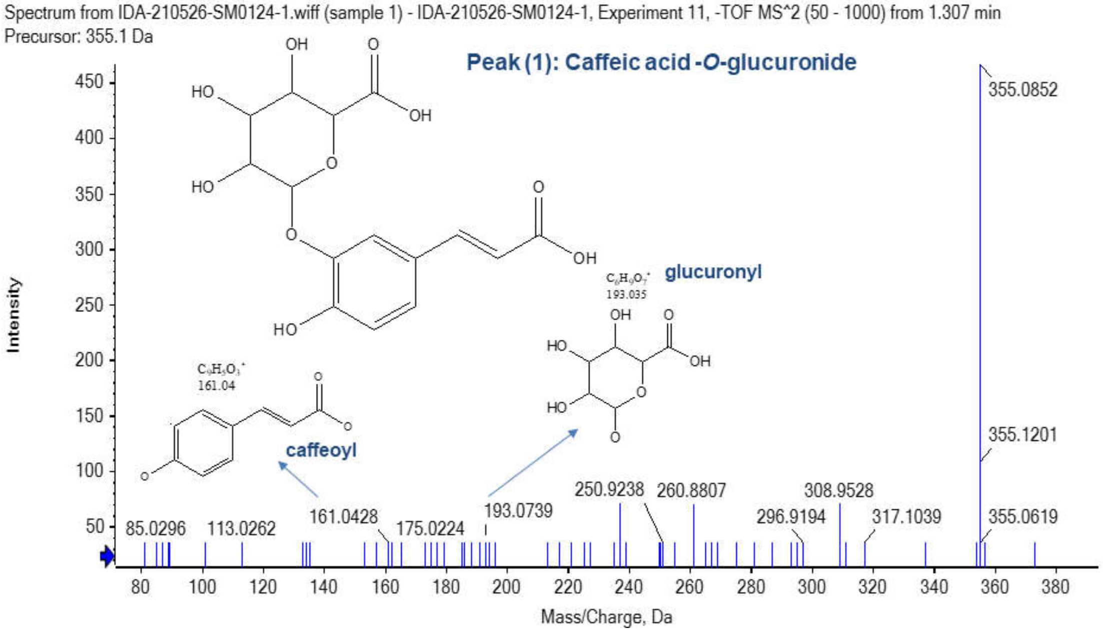

Figure S1: ESI-MS/MS Spectrum of peak (1) in the negative ion mode

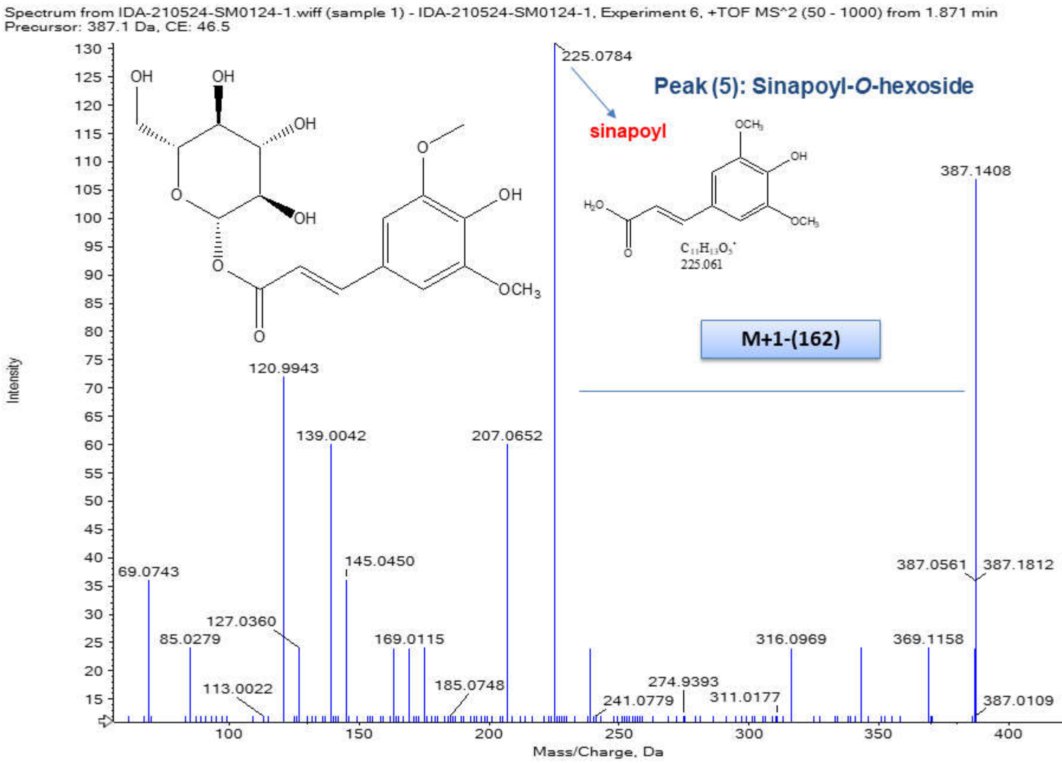

Figure S2: ESI-MS/MS Spectrum of peak (5) in the positive ion mode

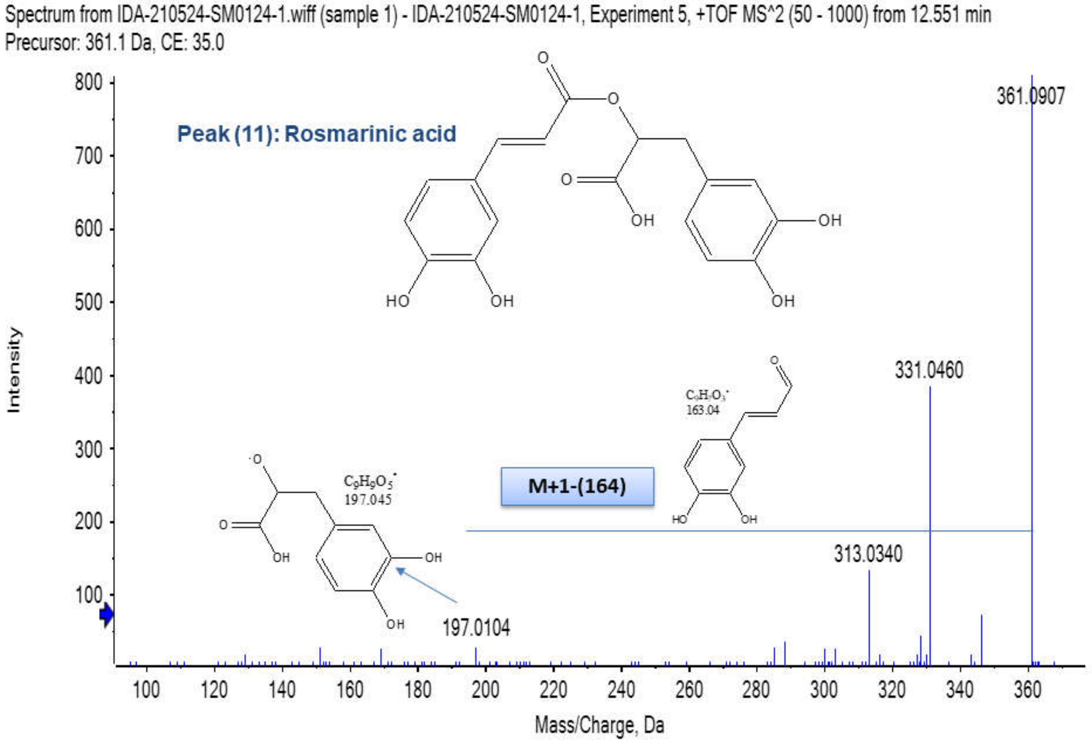

Figure S3: ESI-MS/MS Spectrum of peak (11) in the positive ion mode

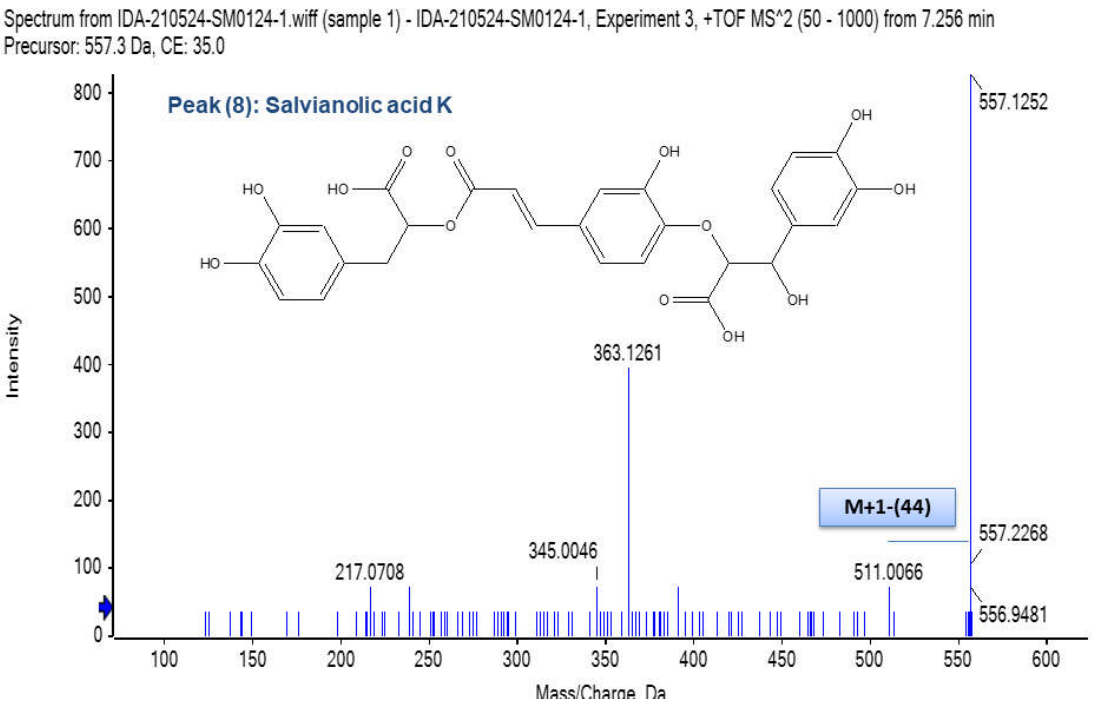

Figure S4: ESI-MS/MS Spectrum of peak (8) in the positive ion mode

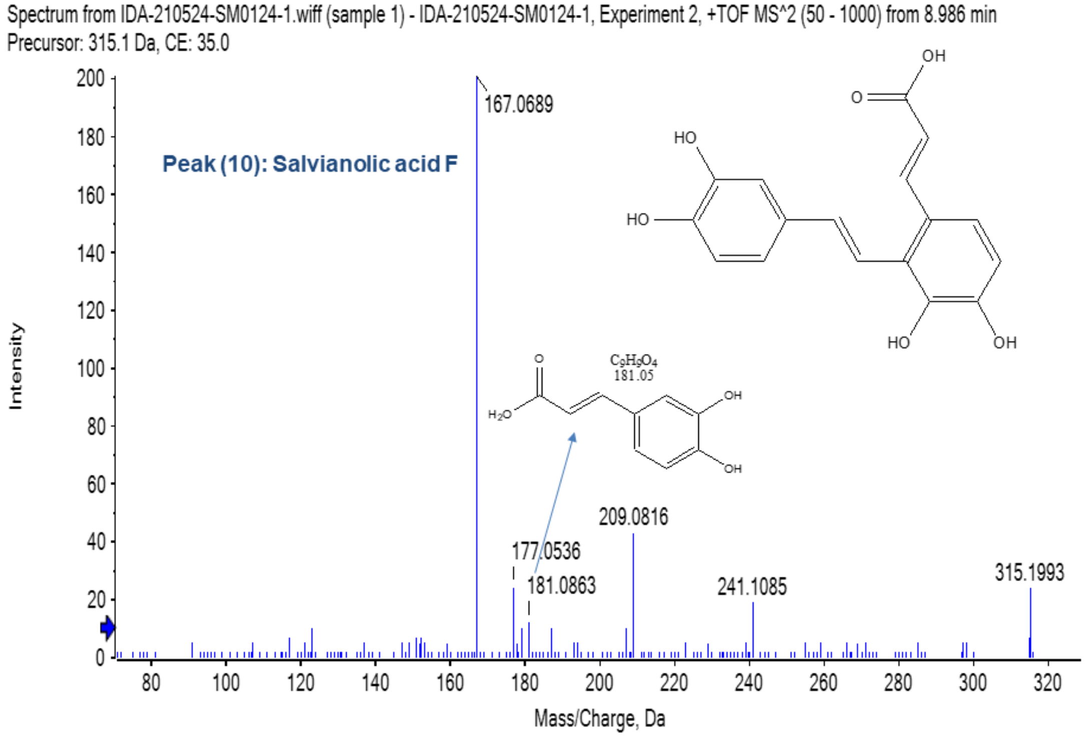

Figure S5: ESI-MS/MS Spectrum of peak (10) in the positive ion mode

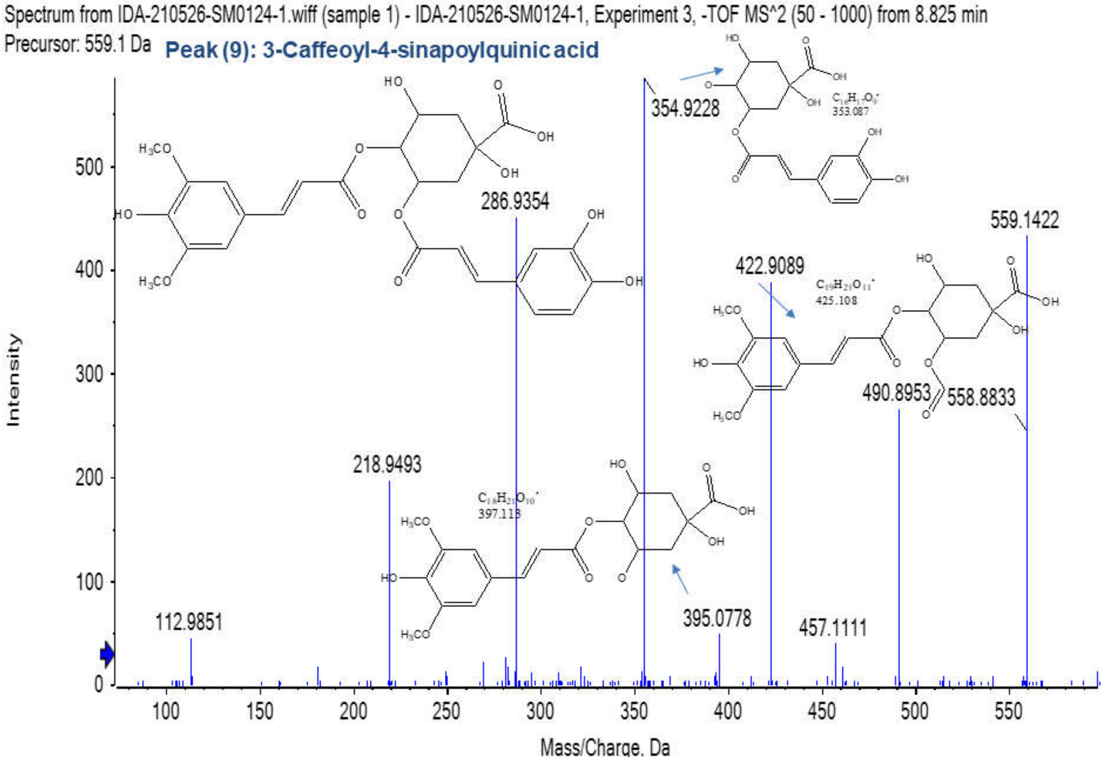

Figure S6: ESI-MS/MS Spectrum of peak (9) in the negative ion mode

Identification of Flavones and derivatives

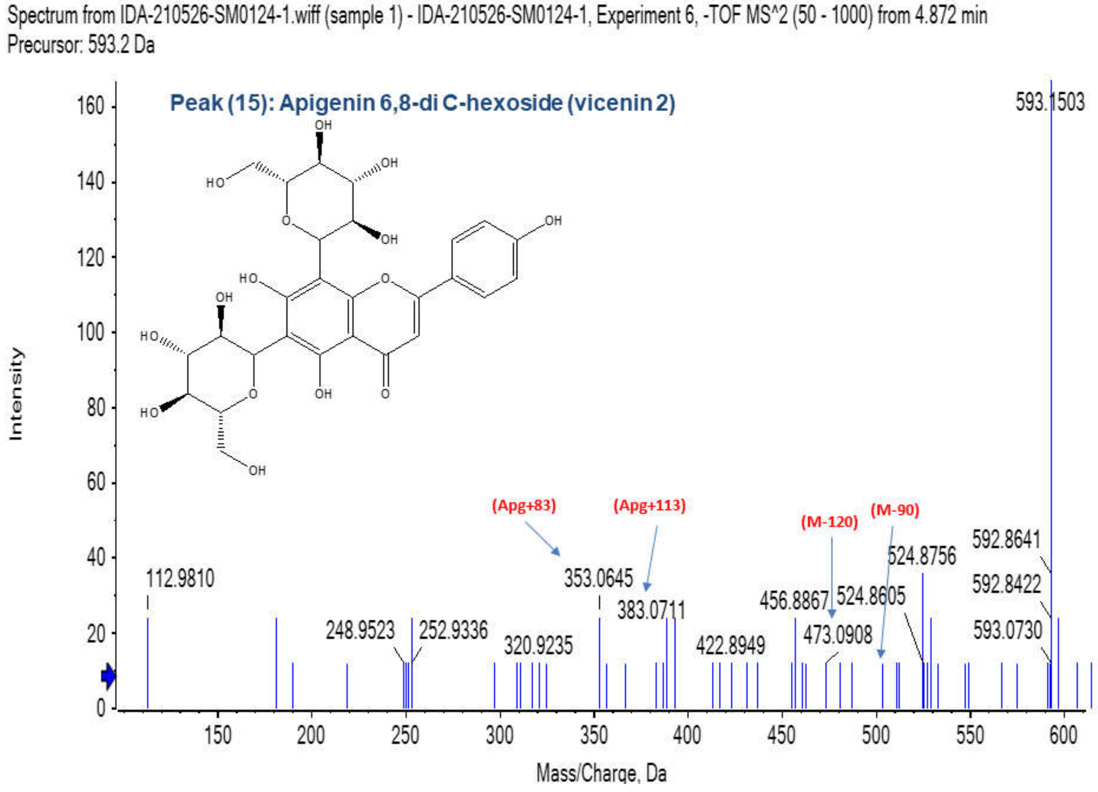

Figure S7: ESI-MS/MS Spectrum of peak (15) in the negative ion mode

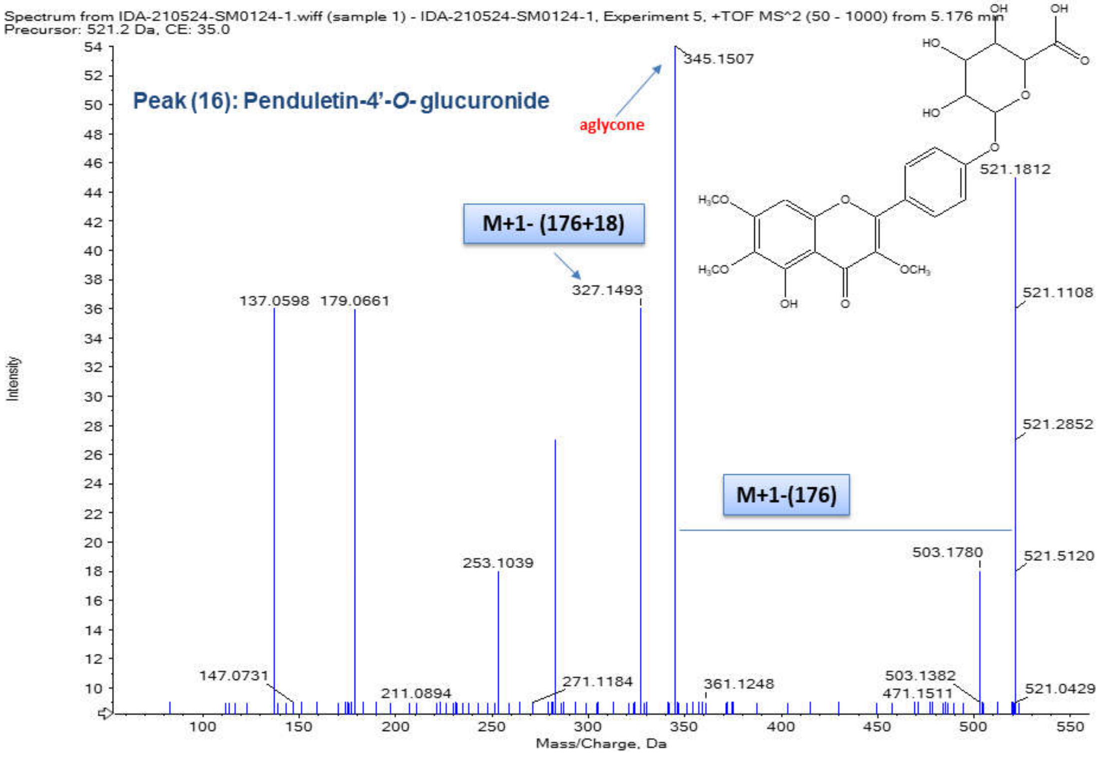

Figure S8: ESI-MS/MS Spectrum of peak (16) in the positive ion mode

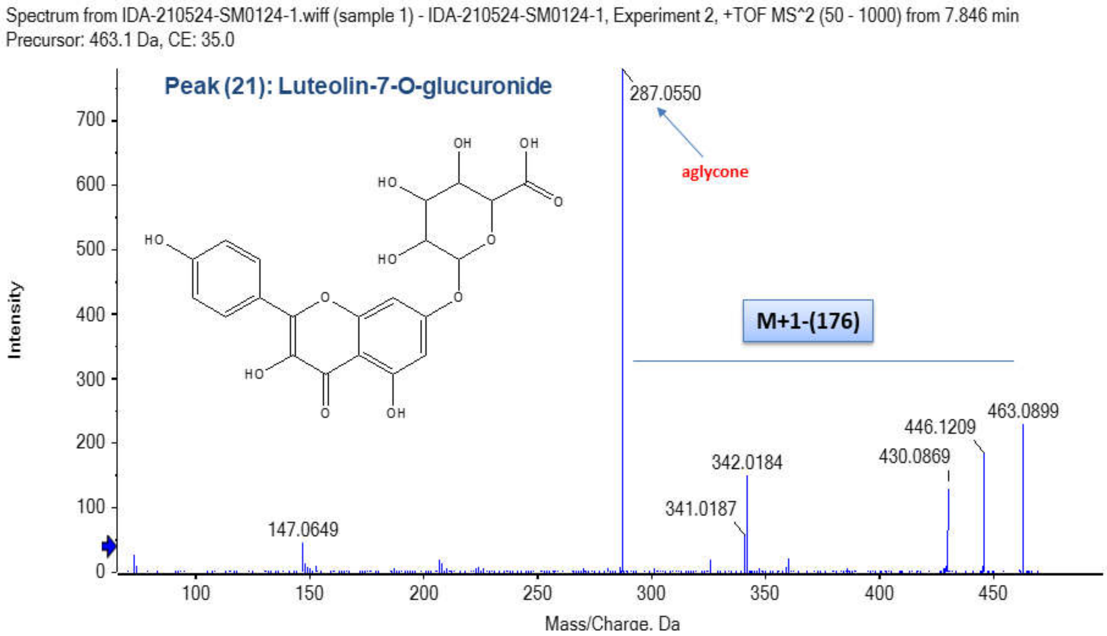

Figure S9: ESI-MS/MS Spectrum of peak (21) in the positive ion mode

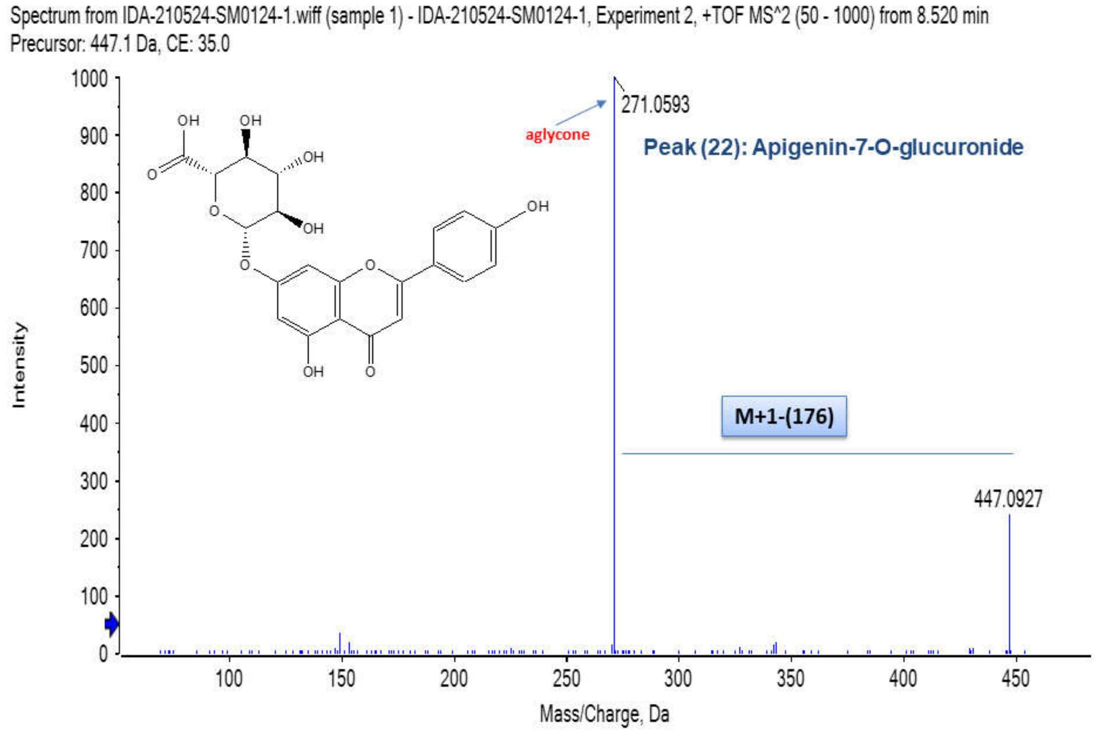

Figure S10: ESI-MS/MS Spectrum of peak (22) in the positive ion mode

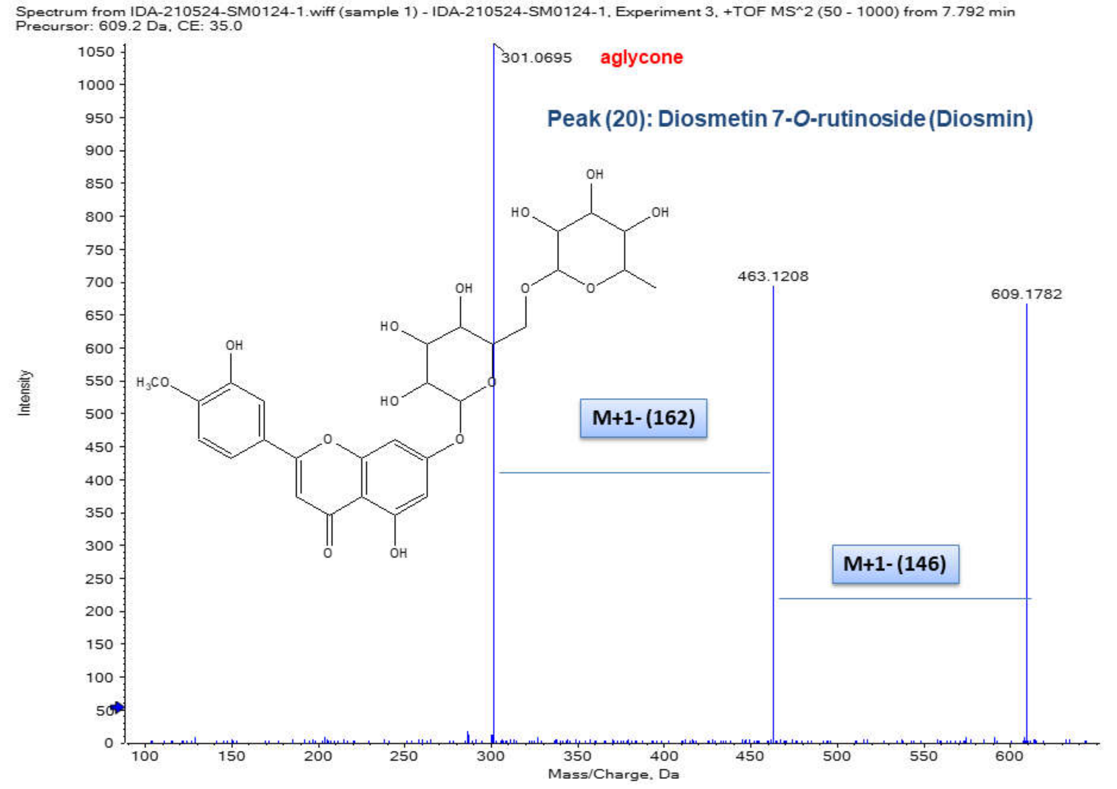

Figure S11: ESI-MS/MS Spectrum of peak (20) in the positive ion mode

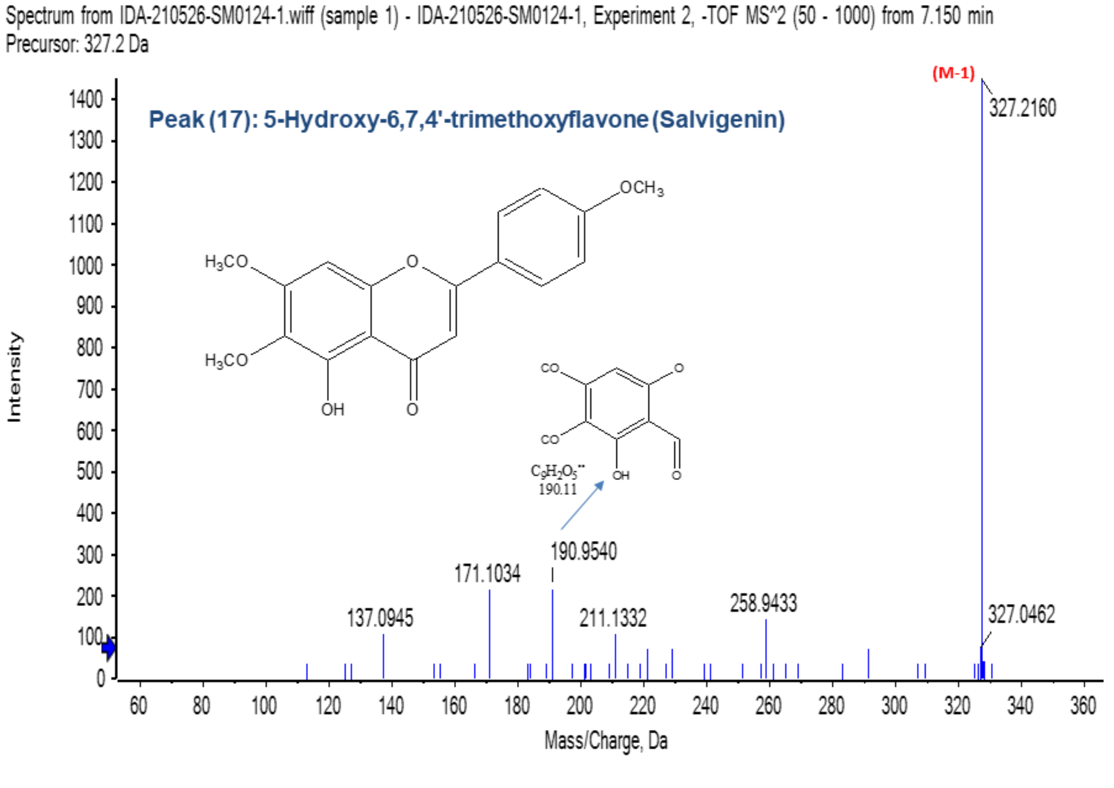

Figure S12: ESI-MS/MS Spectrum of peak (17) in the negative ion mode

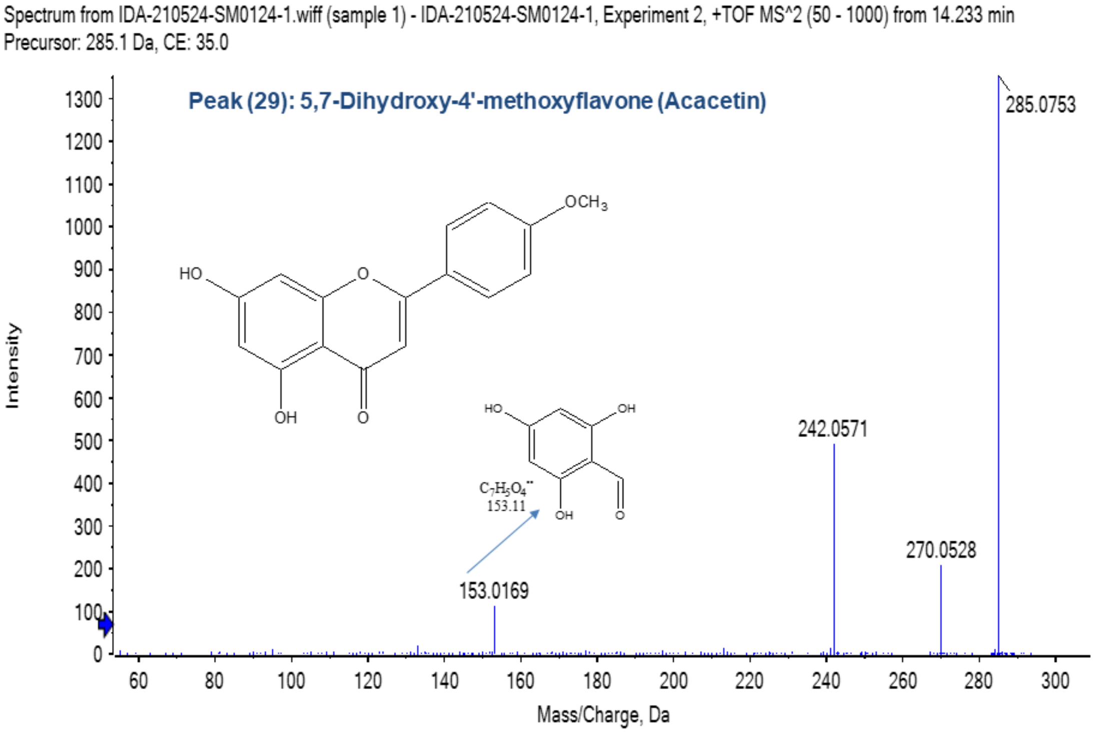

Figure S13: ESI-MS/MS Spectrum of peak (29) in the positive ion mode

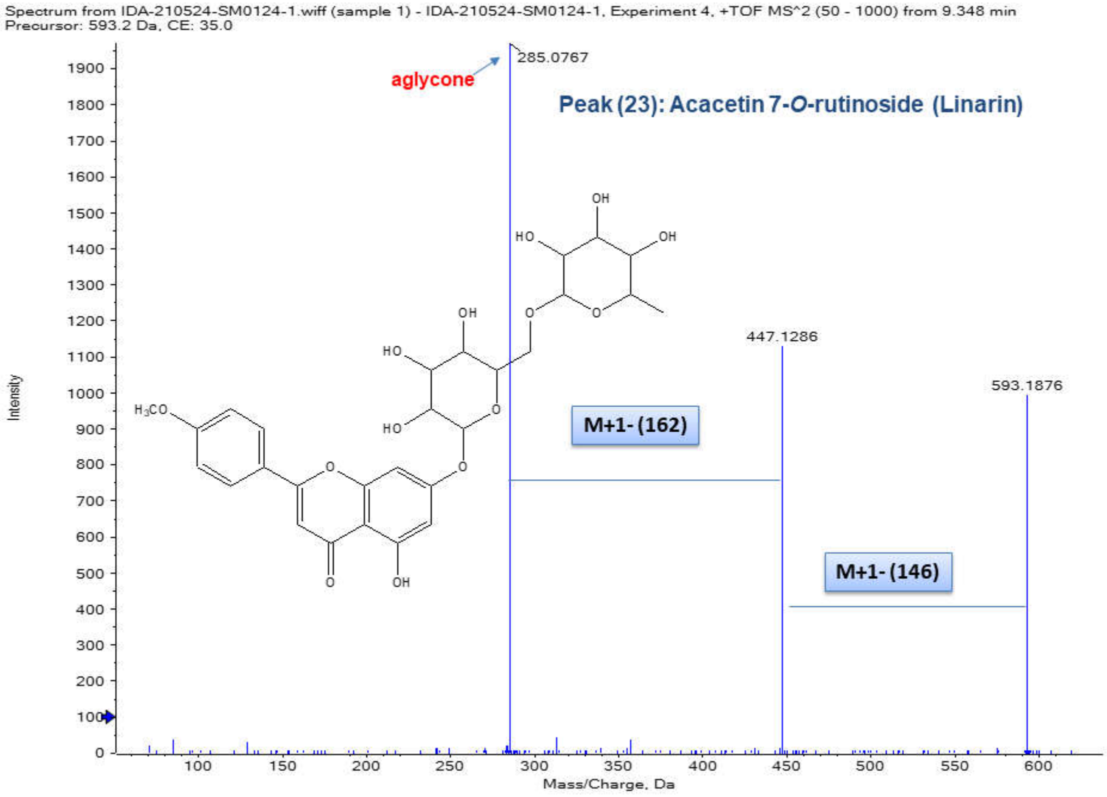

Figure S14: ESI-MS/MS Spectrum of peak (23) in the positive ion mode

Identification of Flavanone & Flavanol derivatives

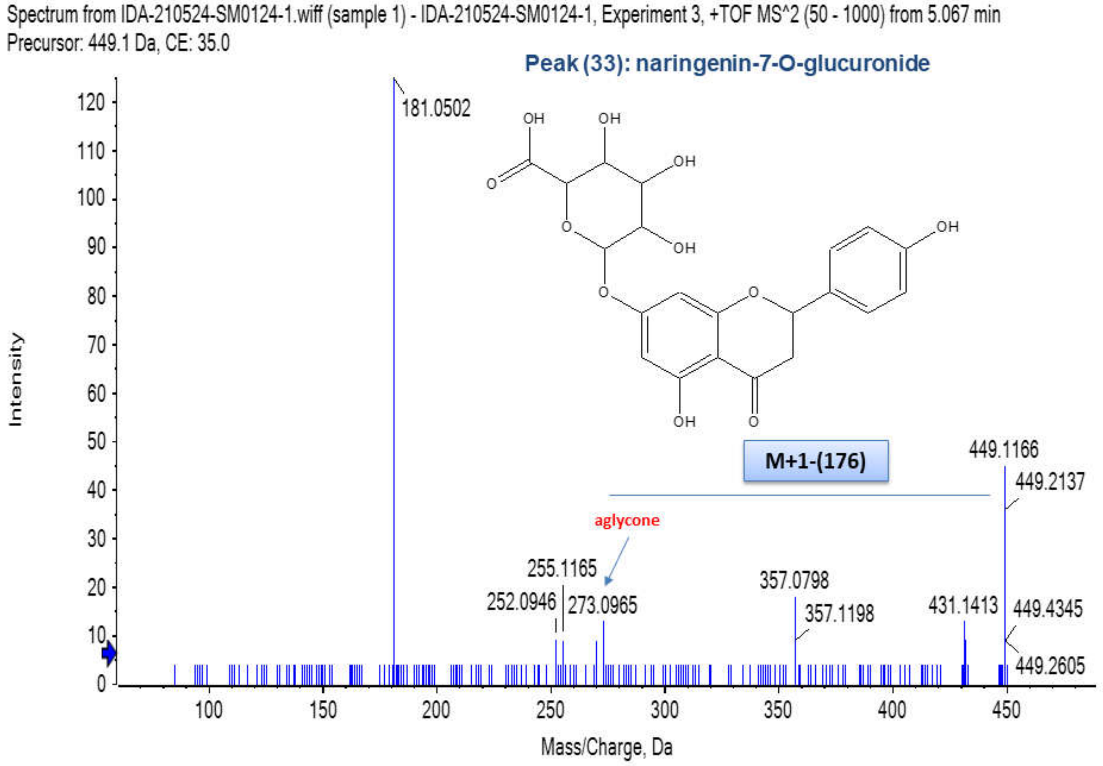

Figure S15: ESI-MS/MS Spectrum of peak (33) in the positive ion mode

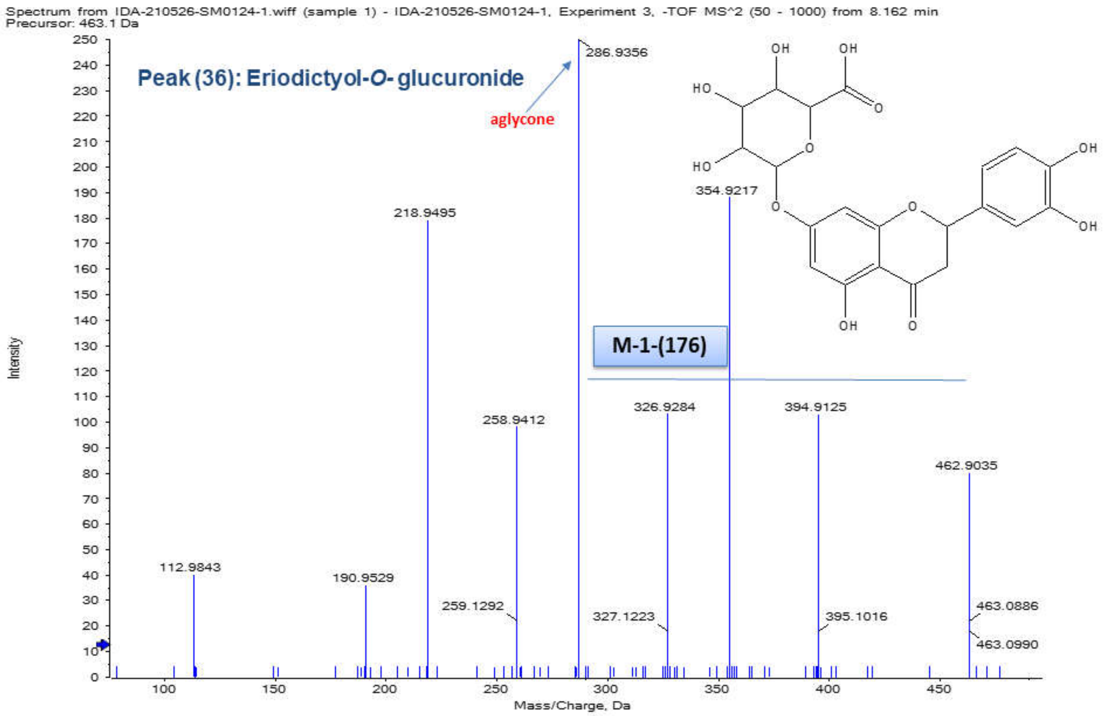

Figure S16: ESI-MS/MS Spectrum of peak (36) in the negative ion mode

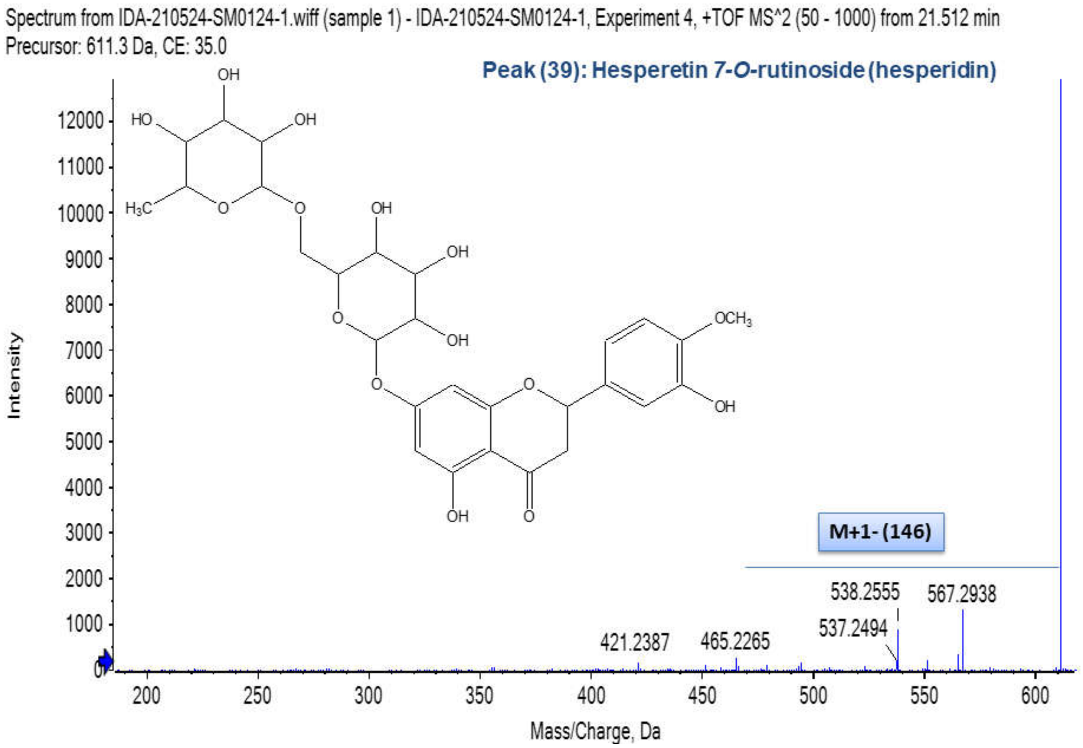

Figure S17: ESI-MS/MS Spectrum of peak (39) in the positive ion mode

Identification of prenyl flavones

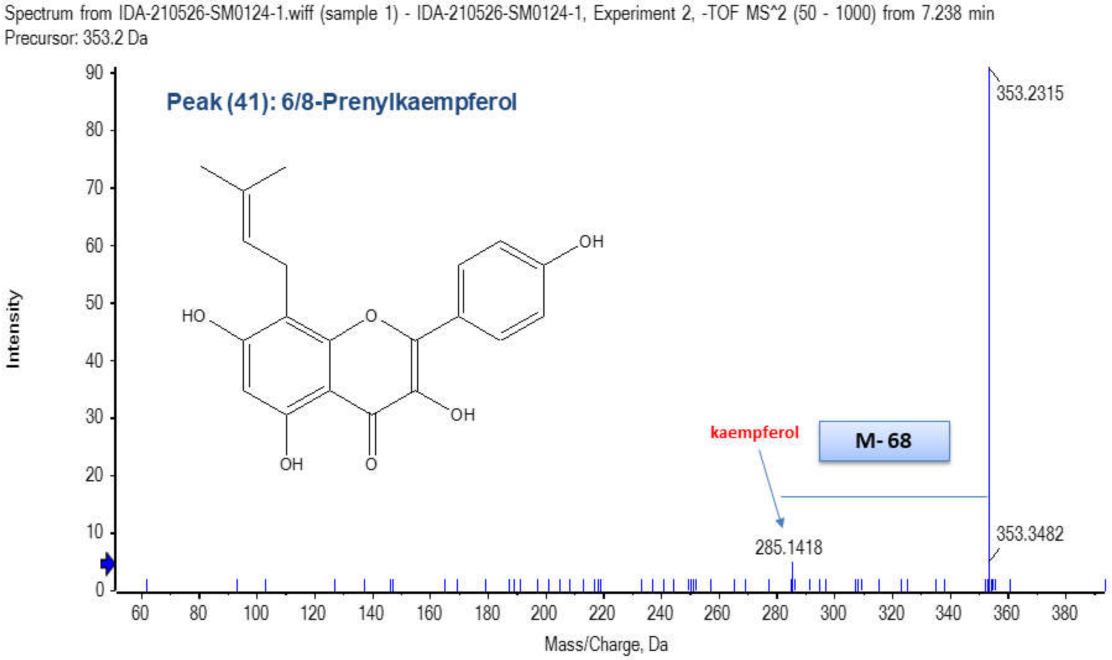

Figure S18: ESI-MS/MS Spectrum of peak (41) in the negative ion mode

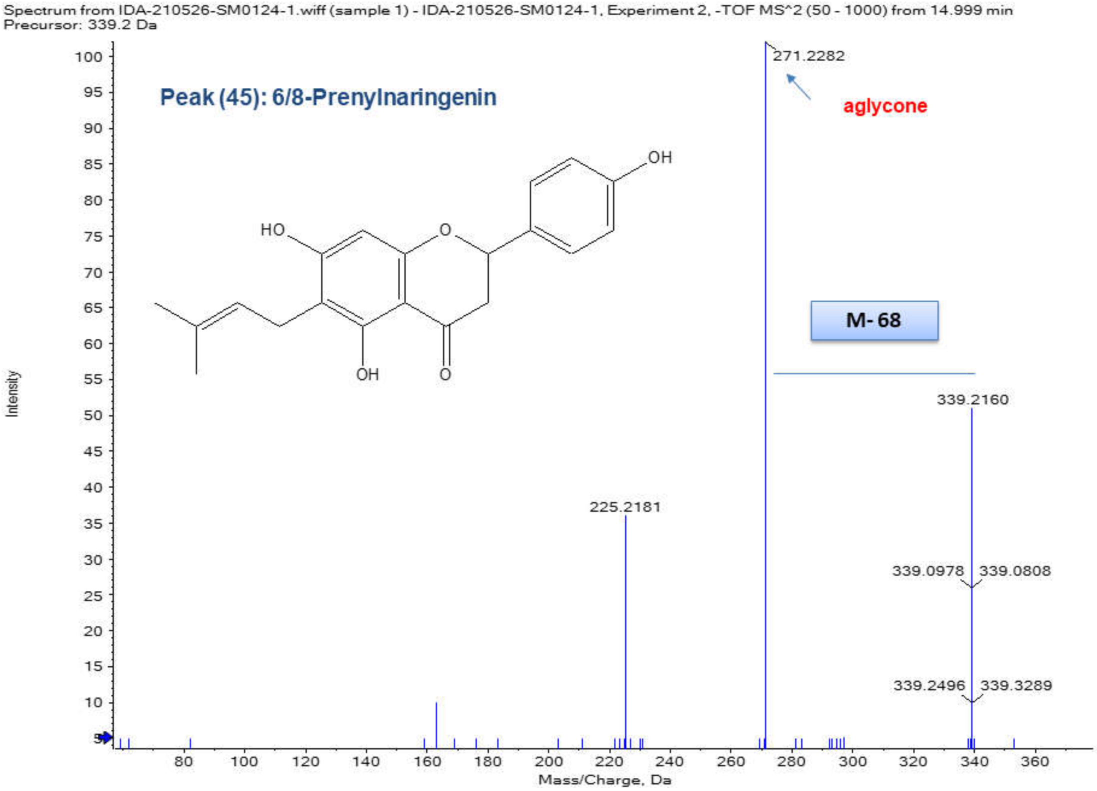

Figure S19: ESI-MS/MS Spectrum of peak (45) in the negative ion mode

Identification of bioflavonoids

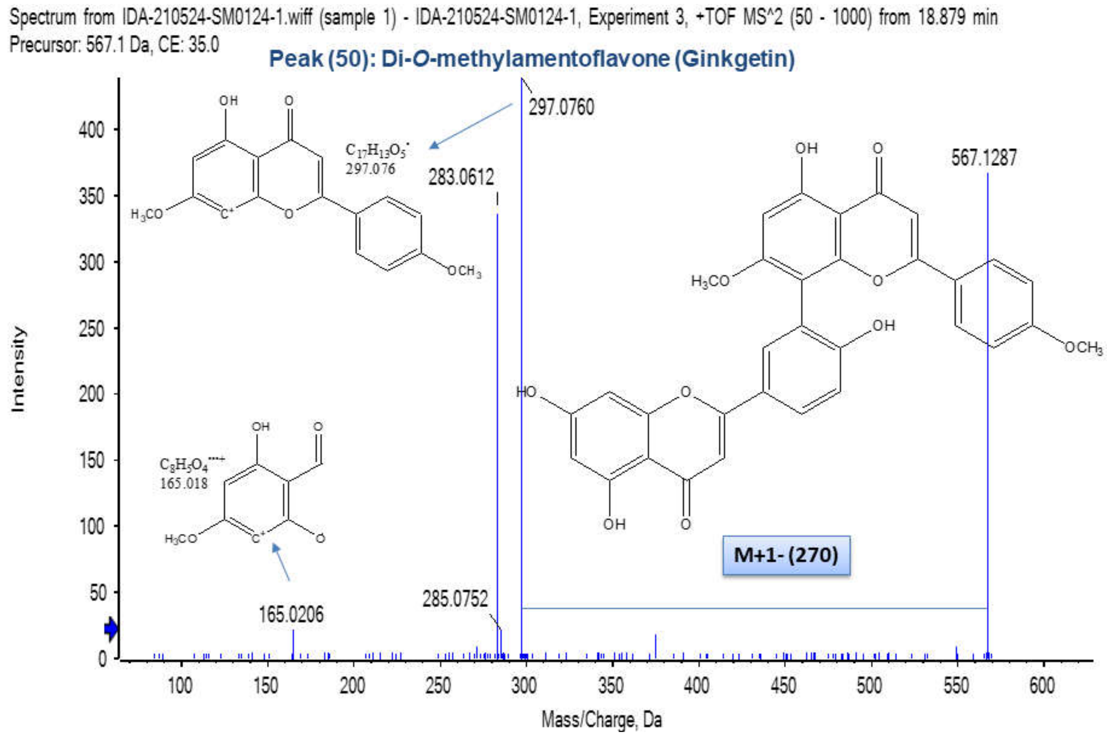

Figure S20: ESI-MS/MS Spectrum of peak (50) in the positive ion mode

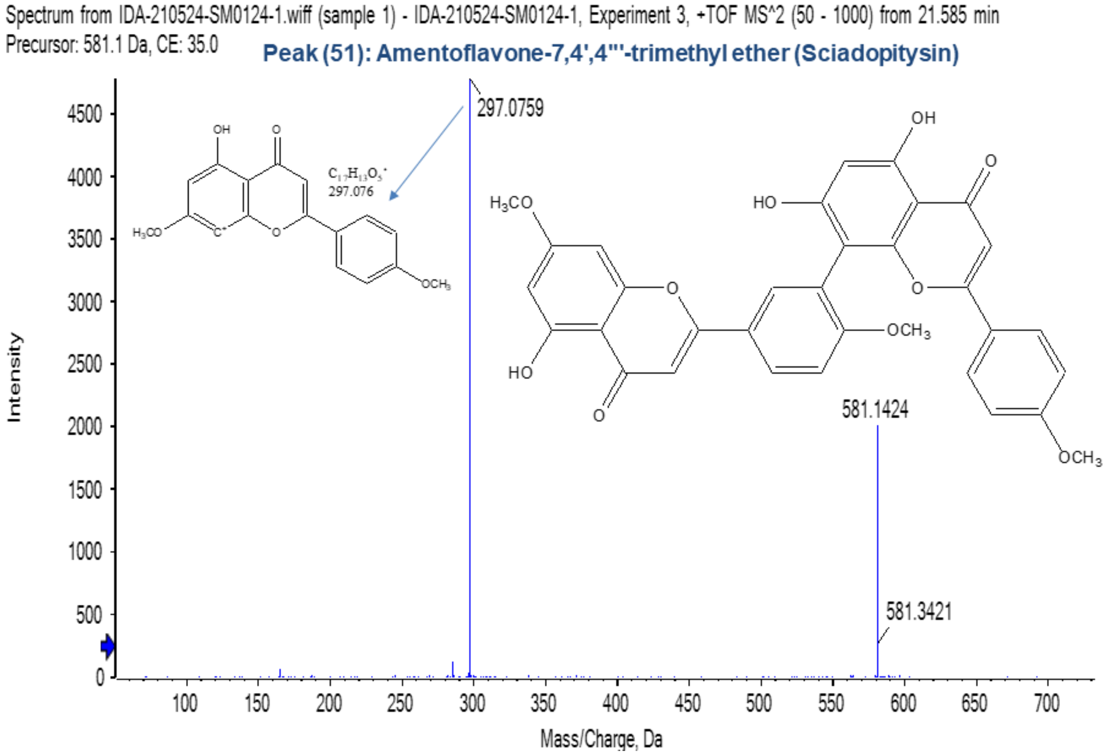

Figure S21: ESI-MS/MS Spectrum of peak (51) in the positive ion mode

Identification of diterpenes

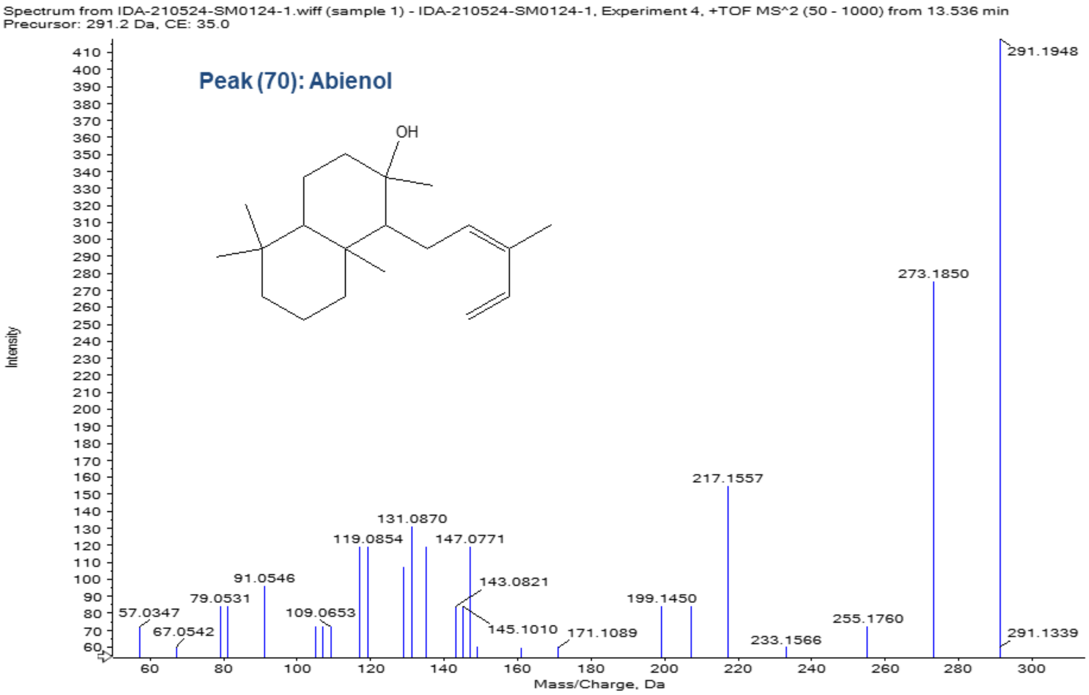

Figure S22: ESI-MS/MS Spectrum of peak (70) in the positive ion mode

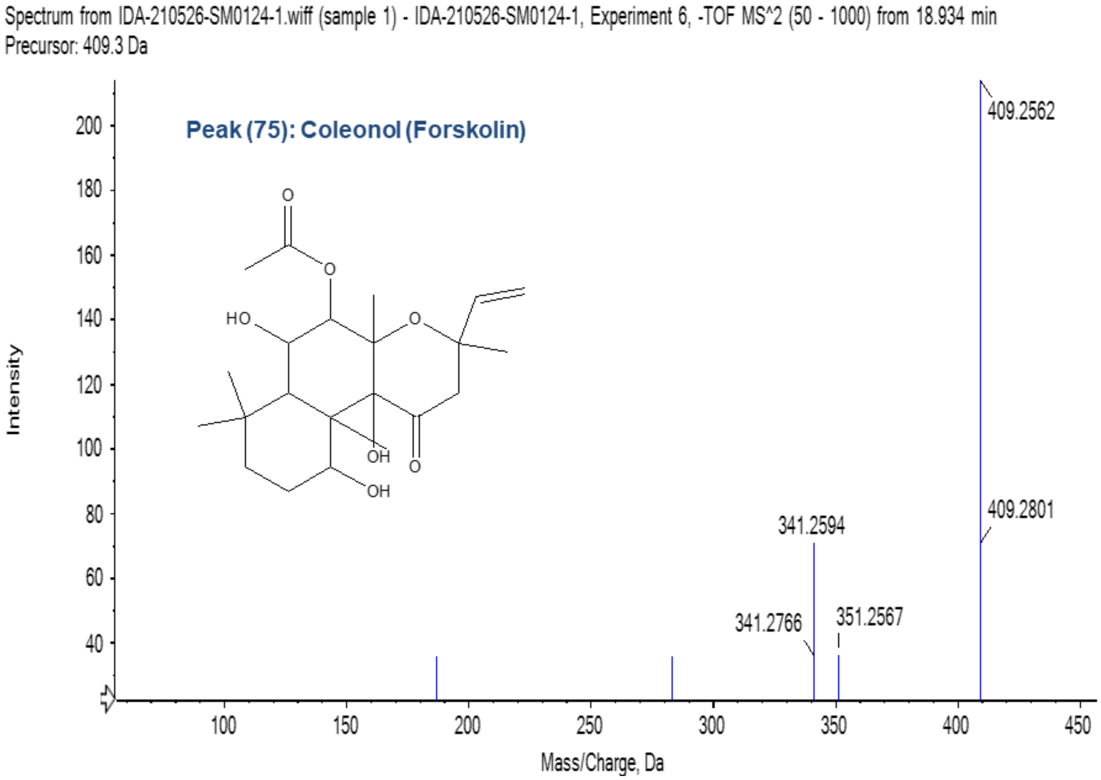

Figure S23: ESI-MS/MS Spectrum of peak (75) in the negative ion mode

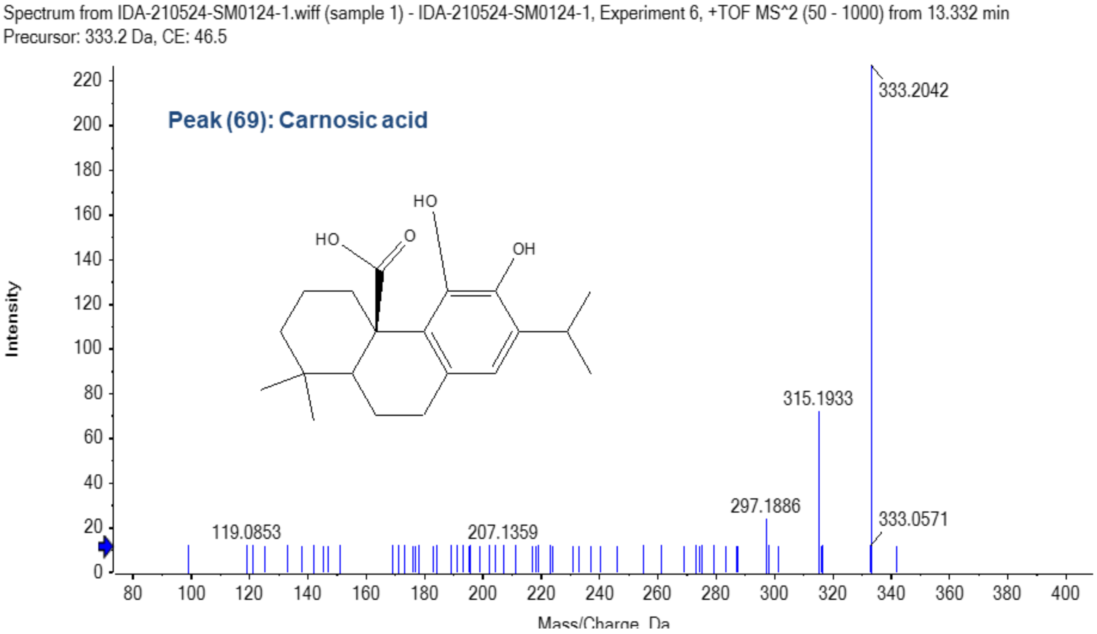

Figure S24: ESI-MS/MS Spectrum of peak (69) in the positive ion mode

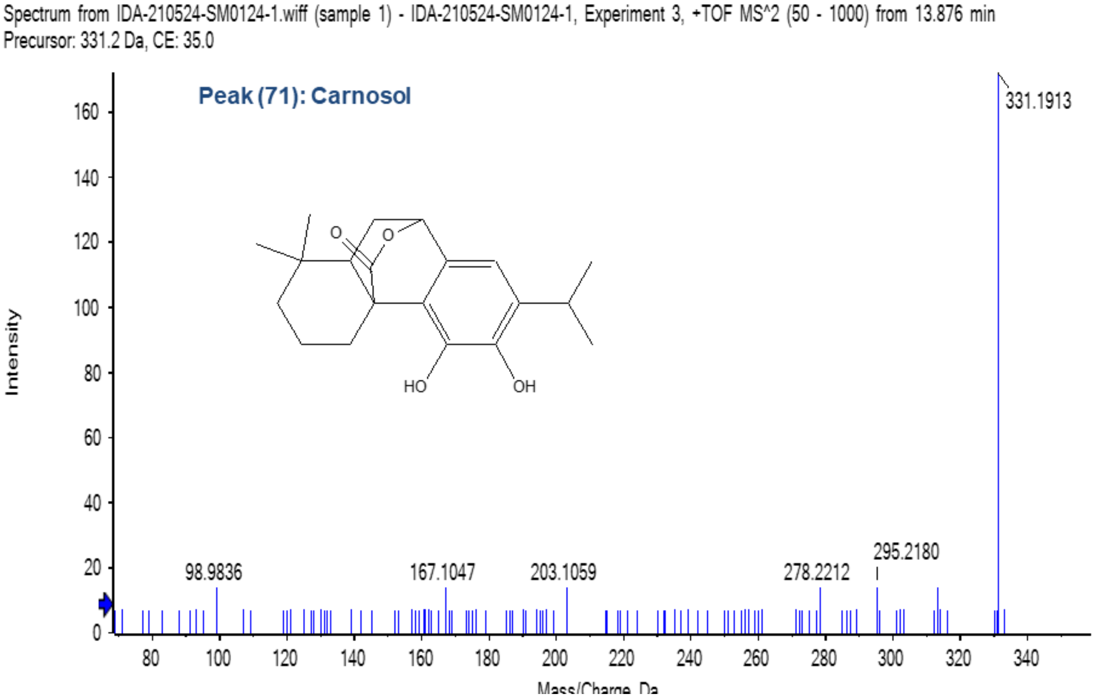

Figure S25: ESI-MS/MS Spectrum of peak (71) in the positive ion mode

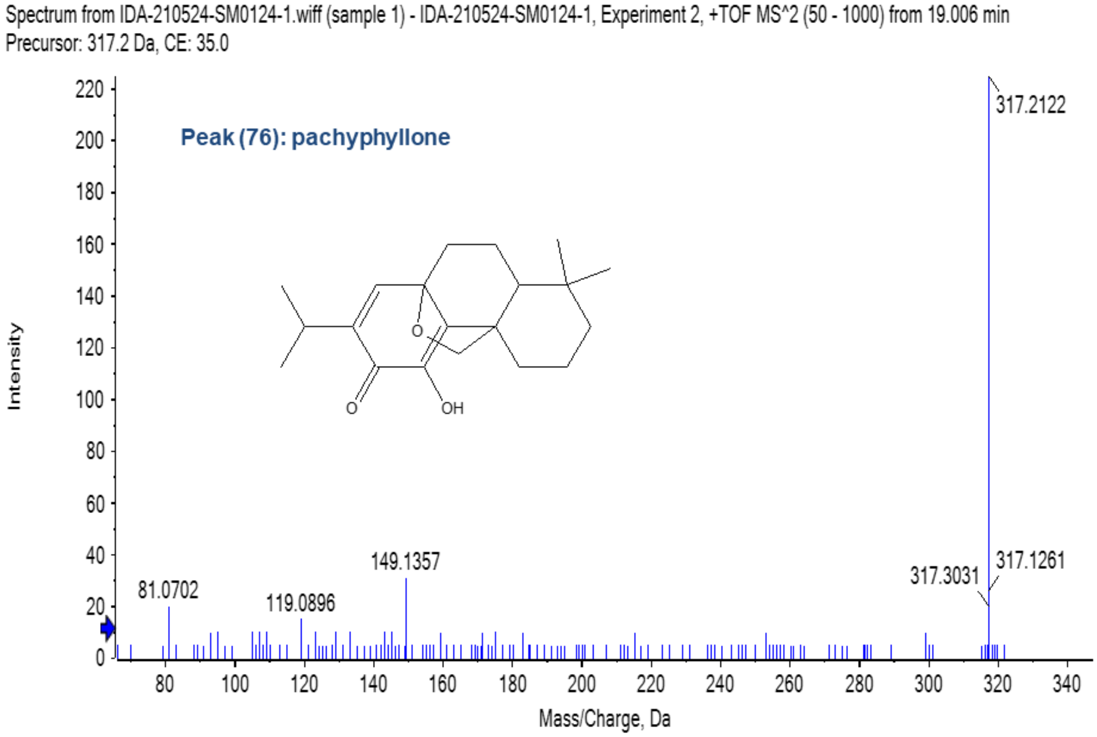

Figure S26: ESI-MS/MS Spectrum of peak (76) in the positive ion mode

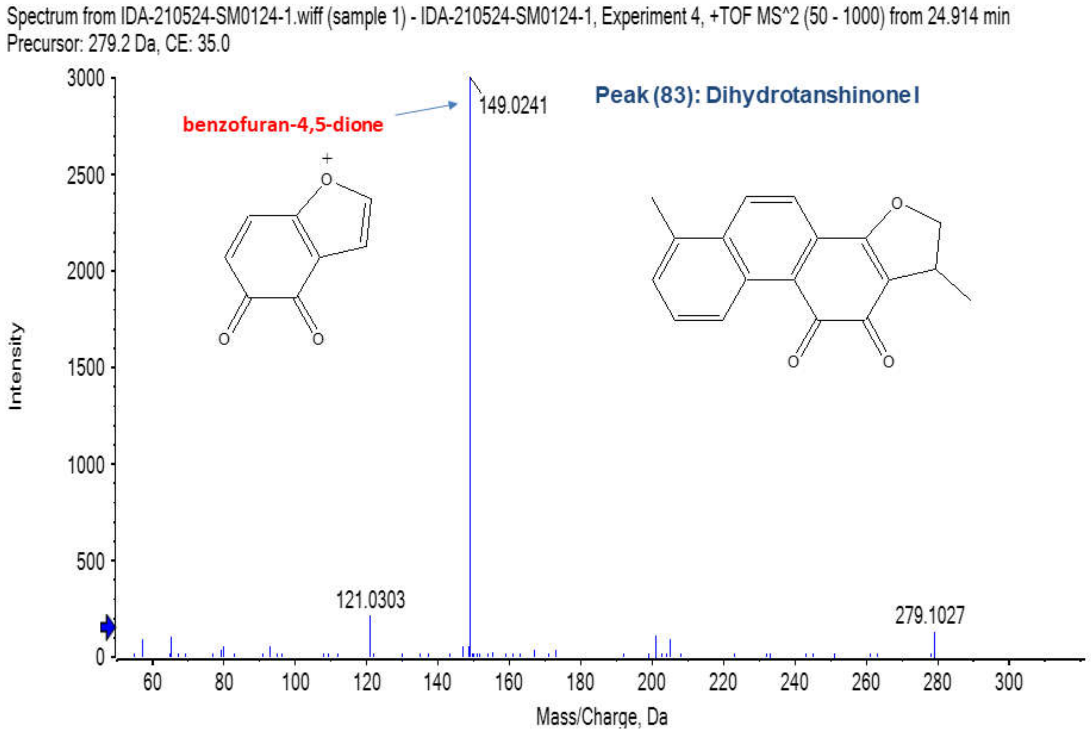

Figure S27: ESI-MS/MS Spectrum of peak (83) in the positive ion mode

Identification of triterpenes

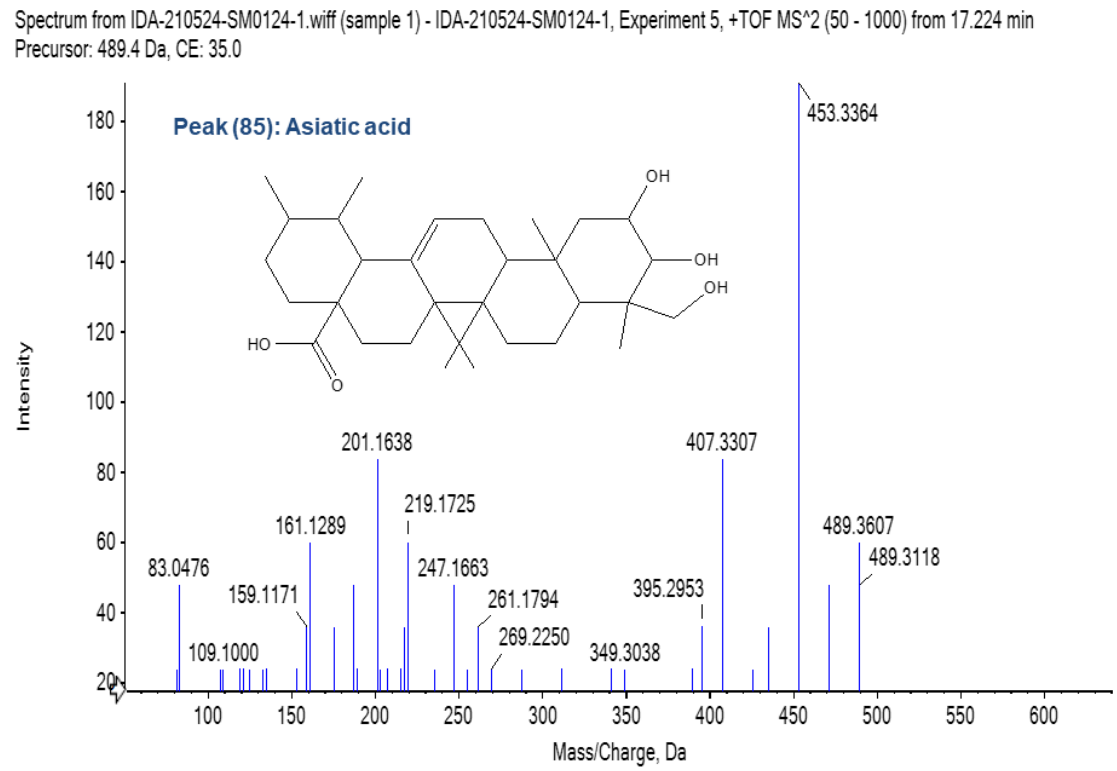

Figure S28: ESI-MS/MS Spectrum of peak (85) in the positive ion mode

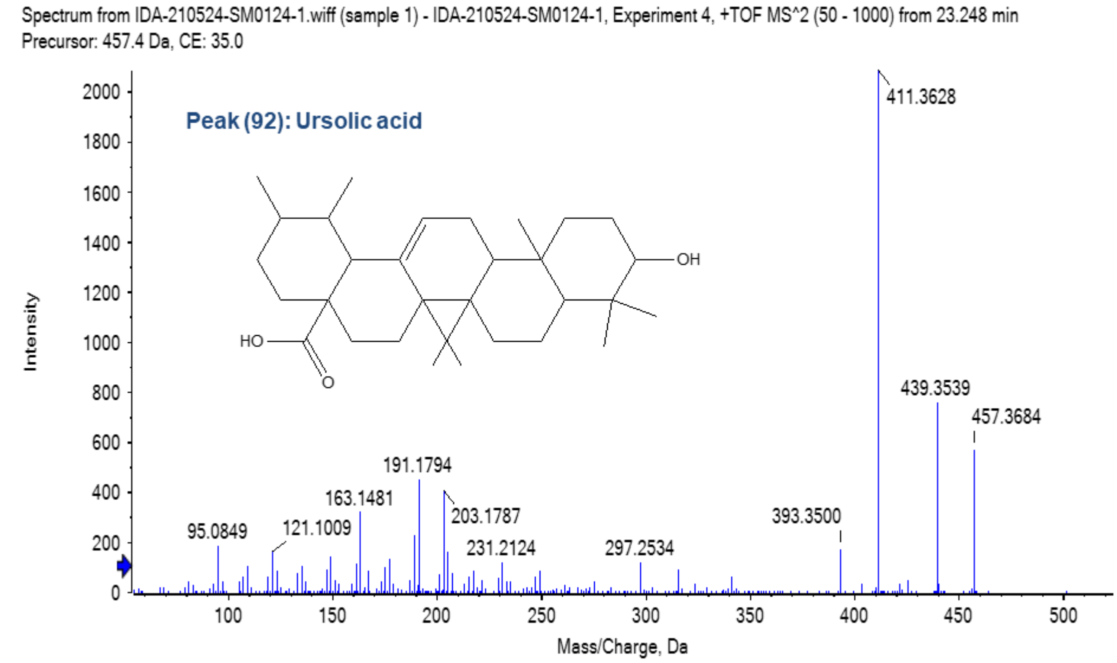

Figure S29: ESI-MS/MS Spectrum of peak (92) in the positive ion mode.

Table S1. The PCR primers used for quantitative PCR analysis.

**MiR -520**	**F: GGCACAAAGTGCTTCCTTTTA**<br>**R: TATGGTTTTGACGACTGTGTGAT**
**MiR -146a**	F: 5′- CTGAGAACTGAATTCCA -3′<br>R: 5′- GAG CAG GCT GGA GAA -3′
**U6**	F: ATTGGAACGATACAGAGAAGATT<br>R: GGAACGCTTCACGAATTTG
